# Case Report: Quantitative and qualitative evaluation for electroacupuncture combined with moxibustion in the treatment of recalcitrant low anterior resection syndrome

**DOI:** 10.3389/fmed.2026.1815559

**Published:** 2026-05-08

**Authors:** Chenxi Hu, Xun Li, Lu Yang, Ruolin Fang, Jinchang Huang, Ming Yang

**Affiliations:** 1 The Third Affiliated Hospital, Beijing University of Chinese Medicine, Beijing, China; 2 Centre for Evidence-Based Chinese Medicine, Beijing University of Chinese Medicine, Beijing, China; 3 Department of Acupuncture and Mini-invasive Oncology, Beijing University of Chinese Medicine Third Affiliated Hospital, Beijing, China

**Keywords:** case report, electroacupuncture, low anterior resection syndrome, moxibustion, rectal cancer

## Abstract

**Background:**

Approximately 60–80% of rectal cancer patients develop low anterior resection syndrome (LARS) following sphincter-preserving surgery, which significantly impacts their quality of life. Existing treatments have various limitations. This case report evaluated the efficacy of electroacupuncture (EA) combined with moxibustion therapy for refractory LARS using a combined quantitative and qualitative approach. The patient was a 55-year-old man who continued to experience severe symptoms (LARS score: 34 points) 8 years after rectal cancer surgery, with sacral nerve modulation treatment proving ineffective.

**Case presentation:**

The patient received EA and moxibustion treatment between 5 December 2024 and 27 December 2024. EA was administered every other day (12 sessions total), targeting the bilateral sacral foramen acupoints: Shangliao (BL31), Ciliao (BL32), Zhongliao (BL33), and Xialiao (BL34). Electrical stimulation lasted 30 min per session with a continuous wave at 50 Hz. Daily moxibustion (23 sessions total) was applied to Shenshu (BL23) and Mingmen (DU4) to warm the kidneys and strengthen the yang. Moxa sticks (30 mm diameter) were held 2 cm above the skin for 45 min per session. After treatment, the LARS score decreased to 11. Daily defecation frequency reduced from 13–15 to 3–4 episodes. Qualitative interview results showed that urgency tolerance increased (from <1 min to 10 min), fecal leakage frequency decreased (from ≥1 daily to <1 weekly), and there was improvement in evacuation difficulties and flatus control. Systemic symptoms (cold extremities, abdominal distension) improved. However, therapeutic effects partially relapsed at 3 months and stabilized at minor LARS levels (score 24) through 1-year follow-up.

**Conclusion:**

By combining quantitative and qualitative outcome assessments, this case report offers initial evidence for short-term symptom management in refractory LARS. High-quality research is still needed to fully assess the efficacy of this treatment.

## Background

Colorectal cancer, the third most common malignant tumor globally (1), has seen improved survival rates through innovative treatment strategies. However, 60–80% of rectal cancer patients develop low anterior resection syndrome (LARS) (2) following sphincter-preserving surgery, characterized by bowel dysfunction, including fecal urgency, increased frequency, evacuation difficulties, and incontinence (3). This chronic condition significantly impairs the quality of life through toilet dependency and psychosocial avoidance behaviors (4). The pathophysiology of LARS involves multifactorial interactions: impaired sphincter complex innervation (5), loss of rectal reservoir function (6), and anorectal motility disorders (7). Current therapies such as pelvic floor biofeedback training, transanal irrigation, and sacral nerve modulation have limitations, including poor adherence and suboptimal cost-effectiveness, highlighting the need for complementary approaches (6, 8). Electroacupuncture (EA) is a therapeutic method that delivers electrical pulses to inserted acupuncture needles. Moxibustion is a therapeutic method of applying burning mugwort (moxa) or other substances near or to particular points or areas of the body to relax, warm, and tonify (9). We present a 55-year-old man with refractory LARS after failed sacral neuromodulation who declined a permanent stoma. An innovative treatment combining EA at Baliao acupoints (BL31–34) with moxibustion at Shenshu (BL23) and Mingmen (DU4) was implemented. This case demonstrates the potential of EA combined with moxibustion to alleviate symptoms, enhance quality of life, and improve negative emotions in patients with refractory LARS. The case study adheres to the Case Report (CARE) Guidelines (10). This study was approved by the Institutional Review Board of the Third Hospital affiliated to Beijing University of Chinese Medicine (no. BZYSY-2022KYKTPJ-07-XZ01).

## Review of the literature

Currently, a few studies suggest that acupuncture can improve LARS. This paper briefly reviews previous studies on acupuncture treatment for LARS and presents its characteristics in Table 1. A pilot study (11) investigated the use of acupuncture, which consisted of a 10-week period on nine patients who were at least 2 years following the surgery with severe LARS. Results showed that the average LARS score decreased from 39 to 30.3 after acupuncture. Six months after acupuncture, the average LARS score was 7.22. Lu-Lu Xu (12) reported significant improvements in the LARS and Wexner scores. At the same time, the EORTC QLQ-C30 score was significantly lower than that in the control group and improved the anal pressure indicators. Li-Zhong Shenl (13) using acupuncture treatment, which was initiated on the first day after surgery, versus a conservative treatment group showed significantly lower Wexner scores, and the therapeutic effect was maintained up to 12 months after surgery. In addition, the levels of serum vasoactive intestinal peptide, motilin, and 5-hydroxytryptamine have changed substantially after treatment. Articles on other diseases show that acupuncture modulates the imbalance between parasympathetic and sympathetic activity (14), and promotes the contraction function of the distal colon (15) to regulate defecation.

**Table 1:** Review of the literature.

Author (Nation) Year	Type of study	Patient	Experimental group	Control group	Acupoints and frequency	Conclusion
Dulskas, Audrius (Lithuania) 2022	Single Arm	ultralow anastomosis (<5 cm from the anal verge); at least 2 years following the surgery; major LARS	EA		CV-3(Zhong ji), CV-6 (Qi Hai), GV-2 (Yao Shu), UB-23 (Shen Shu), UB-32 (Ci Liao), LI-4 (He Gu), ST-36 (Zusanli), KD-3 (Taixi) and Ex-HN-3 (Yintang) once per week for 10 weeks	Acupuncture has a positive and long-lasting effect on bowel function in patients with major LARS following rectal cancer surgery
Lu-Lu Xu (China) 2023	RCT	LARSS>21	EA was added to the control group	routine defecation function training	Baihui (DU20), Yintang (EX-HN 3), Tianshu (ST 25), Qihai (RN 6), Guanyuan (RN 4), Zusanli (ST 36), Shangjuxu (ST 37), Shenshu (BL 23), Pangguangshu (BL 28), Ciliao (BL 32), Zhongliao (BL 33), Huiyang (BL 35) twice a week for 4 consecutive weeks	Electroacupuncture positively impacted LARS following rectal cancer surgery, effectively improving clinical symptoms and anal pressure indicators and patients’ standard of life.
Li-Zhong Shen (China) 2024	RCT	Patients who have just undergone low-tension rectal cancer surgery	Acupuncture was added to the control group	lifestyle interventions, dietary factor changes, levator ani exercises, and oral loperamide treatment	Zusanli (ST 36), Shangjuxu (ST 37), Neiguan (PC 6), Hegu (LI 4) start on the first day after surgery for 4 days	Acupuncture therapy has a positive effect on the rehabilitation of anal function after low-tension rectal cancer surgery; it can effectively help to improve the serum indices of patients, avoid the occurrence of anal incontinence, and reduce the incidence of complications.

Acupuncture has been proven to have beneficial effects on LARS. The previous study’s acupuncture prescription was rooted in the traditional Chinese medicine theory and contained Zusanli (ST 36), Shangjuxu (ST 37), Tianshu (ST 25), and others. Based on clinical experience treating LARS, our team summarizes (16) that the Baliao acupoints can regulate the opening and closing of the anus. Moreover, our team’s previous articles have demonstrated the beneficial effects of 6 months of acupuncture (Baliao acupoints) in patients newly diagnosed with LARS after surgery who did not undergo any interventions (17). The challenge in this case lies in the patient’s stubborn and refractory LARS symptoms, persisting for 8 years post-surgery despite failed sacral nerve treatment. Additionally, due to transportation constraints, the patient could only receive acupuncture treatment at our hospital for 1 month. Therefore, based on the above considerations, we decided to combine moxibustion with the foundational EA treatment at the Baliao acupoints. This approach aims to explore the efficacy of short-term, high-frequency combined therapy for patients with refractory LARS.

## Case presentation

A 55-year-old man was diagnosed with mid-upper rectal cancer in March 2016. He underwent neoadjuvant radiotherapy followed by rectal anterior resection with terminal ileostomy in June 2016. Postoperative pathology confirmed low differentiated adenocarcinoma with partial response to neoadjuvant radiotherapy, staged as ypT3N0Mx. This patient was predicted to have major LARS by the pre-operative LARS (POLARS) score prediction online tool (18)^.^ From July to December 2016, he received adjuvant chemotherapy. Ileostomy closure was performed in February 2017. Post-reversal, the patient developed fecal incontinence, increased bowel frequency (15–20 times/day), loose stools, and incomplete evacuation.

**Figure 1:**
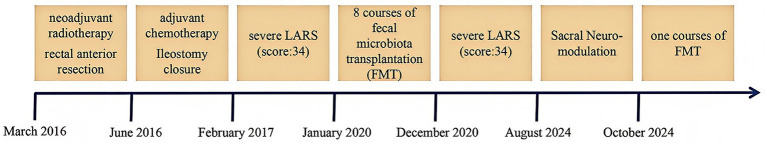
Patient’s previous treatment history.

Between 2020 and 2021, he underwent eight courses of fecal microbiota transplantation (FMT). Each treatment transiently reduced bowel frequency to 7–8 times/day for 2 weeks, but symptoms recurred within 3 months. A colonoscopy in April 2024 revealed post-surgical changes and radiologic colitis. In August 2024, the patient accepted temporary sacral nerve stimulation (SNS) lead implantation, resulting in reduced bowel frequency (4–5 times/day) for 4 days, followed by relapse to pre-treatment levels. The patient declined implantation of a permanent SNS device. Repeat FMT in October 2024 showed no improvement. As of 5 December 2024, the patient presented with persistent fecal incontinence, urgent defecation (particularly triggered by food intake), frequent bowel movements (13–15 times/day), small-volume loose stools, fecal leakage during the day or night, incomplete evacuation, intermittent left abdominal distension/pain, cold extremities, and reduced oral intake. The patient’s previous treatment history is shown in Figure 1.

**Figure 2:**
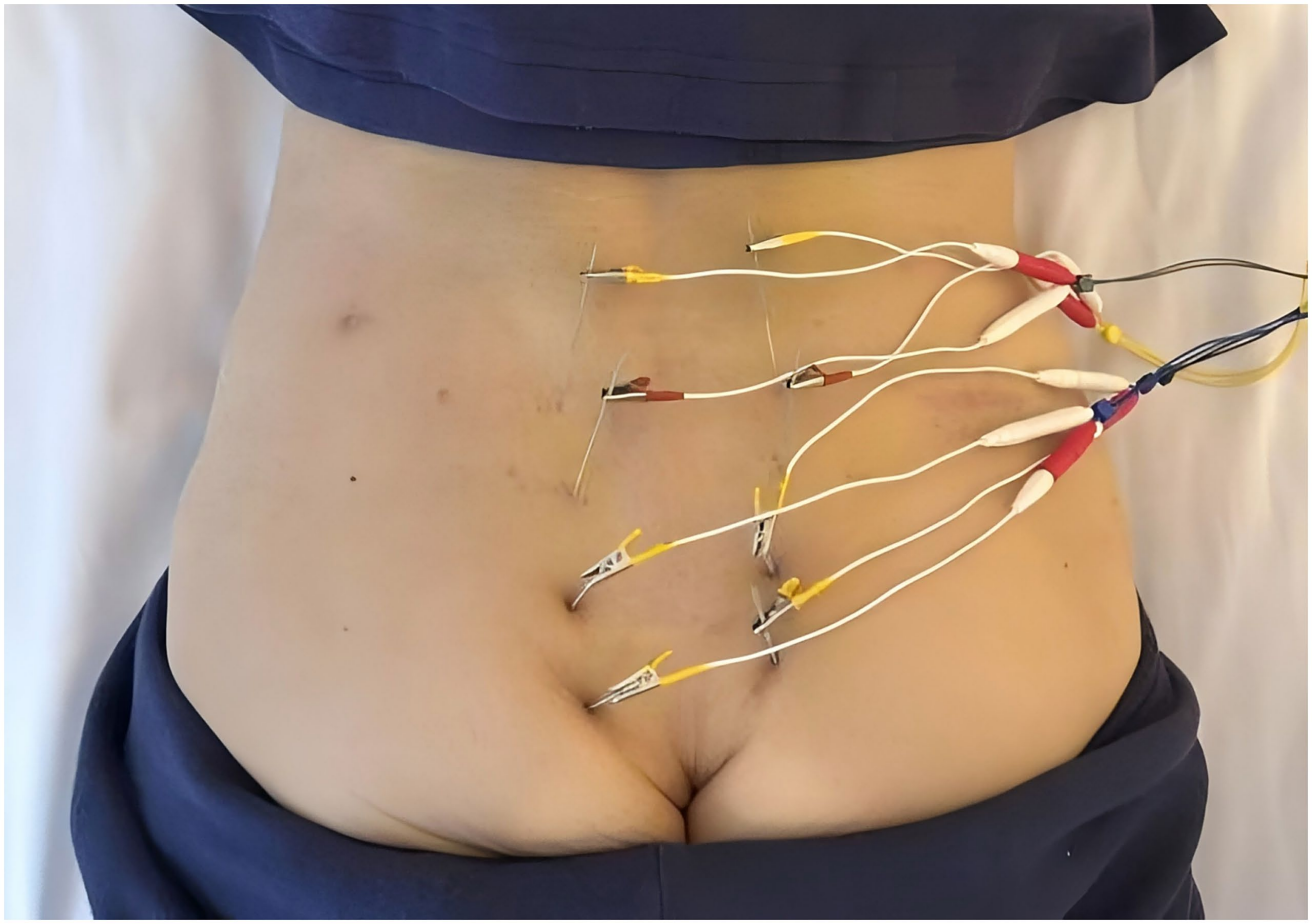
Photo of EA.

From 5 December 2024 to 27 December 2024, the patient received combined EA and moxibustion therapy. EA was administered every other day for 12 courses. The acupoints selected were the eight sacral foramina: Shangliao (BL31), Ciliao (BL32), Zhongliao (BL33), and Xialiao (BL34) bilaterally. Deep insertion was achieved using 0.30*75 mm needles, with angles varying by point, which was based on our previous clinical experience (16): BL34 vertically; BL33 obliquely (~70°) inferomedially; BL32 obliquely (~50°) inferomedially; BL31 obliquely (~30°) inferomedially. Minor needle manipulation (rotation, lifting-thrusting) elicited de-qi sensation, characterized by mild soreness, numbness, or distention radiating towards the anus. After needle insertion, minor amplitude manipulations, including rotation and lifting-thrusting, were performed to achieve a de-qi sensation. Electrodes of the EA apparatus were then transversely connected to the needle handles of bilateral BL31 to BL34. Electrical stimulation lasted 30 min, using continuous-wave at 50 Hz. The current intensity was maintained at 1–5 mA (adjusted to the patient’s tolerance to produce a radiating sensation without pain or discomfort). EA is illustrated in Figure 2. Moxibustion was performed daily for 23 courses. Moxibustion acupoints included Shenshu (BL23) and Mingmen (DU4). Suspended moxibustion was applied using 30 mm diameter moxa sticks. The burning end was maintained 2 cm above the skin surface. The duration was 45 min per acupoint per session, adjusted to the patient’s thermal tolerance.

We also provided dietary and lifestyle guidance to patients during the treatment period. The patients were instructed to avoid spicy and greasy foods, cold beverages, alcoholic drinks, nuts, and sweet foods, including fruits, honey, cakes, and other sweets. A low-fat and low-protein diet was recommended. A moderate intake of coarse fiber foods was suggested. Patients were advised to engage in appropriate physical activity, maintain adequate sleep, and maintain regular routines. Moreover, encourage participation in a variety of social events if the patient pays excessive attention to defecation.

## Results

This case study combines quantitative and qualitative methods of outcome evaluation. The primary objectives were: (1) Triangulation: Quantitative research provides objective, quantifiable efficacy data through standardized questionnaires to test the effectiveness of acupuncture interventions. Qualitative research obtains in-depth, contextual experiential descriptions through semi-structured interviews to elucidate and corroborate quantitative findings. The convergence and integration of both approaches enhances the reliability and validity of research conclusions. (2) Complementarity of Breadth and Depth: While quantitative questionnaires offer objectivity, they may fail to capture the full complexity of LARS symptom fluctuations and the subtle nuances of their impact on daily life. Qualitative interviews effectively address this limitation by sensitively exploring symptom changes, coping strategies, and psychosocial impacts beyond quantitative measures. They deliver a comprehensive and holistic efficacy assessment, revealing how acupuncture works and its practical significance in patients’ lives. (3) Exploration and Optimization: Beyond pure efficacy assessment, this study also explores patients’ subjective experiences and perceptions of acupuncture treatment. It examines their acceptability and preferences regarding treatment protocols (e.g., needle sensation, session design, and frequency), thereby providing preliminary evidence for optimizing patient-centered, individualized clinical acupuncture treatment plans.

For quantitative analysis, two scales specifically designed for LARS patients were used to evaluate bowel function before and after acupuncture treatment: the LARS score questionnaire and the MSKCC-BFI questionnaire (19). The EORTC QLQ-C30 questionnaire, a quality-of-life scale tailored for cancer patients, was used to assess patients’ quality of life.

The LARS score is a simple tool for quick clinical evaluation of the severity of LARS and is the only tool globally used explicitly for assessing bowel dysfunction after low anterior resection (LAR) for rectal cancer. The total score ranges from 0 to 42, and is divided into 0 to 20 (no LARS), 21 to 29 (minor LARS), and 30 to 42 (major LARS) (20). We utilized the Chinese-translated and validated version, which has been demonstrated to be adapted in clinical and research settings in the Chinese population (21). LARS score assessments were conducted prior to treatment initiation and after every three treatment sessions.

The MSKCC-BFI questionnaire comprises 18 items across three subscales: urgency, dietary influence on bowel movements, and abnormal bowel sensations. All questions can be evaluated in five different frequency ranges. The total score is calculated by summing all 18 items. Higher scores indicate better bowel function (22). The MSKCC-BFI questionnaire was assessed before treatment and after 12 treatments.

The EORTC QLQ-C30 questionnaire comprises 30 questions, including 5 functional scales, 9 symptom scales, and a global health status. The functional scales and global health status range from 0 (worst function) to 100 (best function). The symptom scales range from 0 (best function) to 100 (worst function) (23). The EORTC QLQ-C30 questionnaire was assessed before treatment and after 12 treatments.

Follow-up was conducted by telephone, with a brief conversation with the patient after completion of the LARS questionnaire.

We used a qualitative research design using semi-structured interviews to gather information. This process included recording interviews, transcribing transcripts, and conducting thematic analysis. The interviewer was the first author of this article and also the patient’s acupuncturist. While potential bias may exist due to prior therapeutic interactions, the author’s deep understanding of the patient’s condition enabled a more comprehensive and in-depth exploration. The patient agreed to a face-to-face, in-depth, semistructured qualitative interview lasting approximately 30 min after completing 12 treatment sessions, with the interview outline shown in Table 2. The patient was required to recall symptoms before and after the treatment, as well as the feelings during the treatment. The interview data were transcribed verbatim by the first author and checked against the recordings. Then, two authors coded line by line and generated preliminary themes using NVivo 12 (24), which were ultimately agreed upon by all authors.

**Table 2:** Semistructured qualitative interview outline.

Number	Question
1	What discomfort did you experience before receiving treatment?
2	Do these discomforts affect your psychology, daily life, and social activities, and what kind of impact do they have?
3	What symptoms have improved after receiving treatment? How are you feeling now?
4	What changes does treatment bring to your psychology, daily life, social activities, etc.?
5	What are your feelings during the process of receiving electroacupuncture treatment?
6	What do you think are the advantages and disadvantages of acupuncture and moxibustion compared with the treatment you have received before?

Following treatment, the patient exhibited marked improvement in the LARS score, decreasing from 34 to 11 points. Fecal incontinence symptoms are completely controlled. Bowel movements are stable three times a day. Defecation difficulties have improved, eliminating the need for repeated bowel movements within an hour. Although the urgency persists, its frequency has significantly decreased.

The patient reported not strictly adhering to dietary and lifestyle guidelines during follow-up. During the 6 months following the intervention, patients reported continuing moxibustion at home once or twice a week; thereafter, they suspended moxibustion for personal reasons. At 1-month follow-up, the LARS score remained at the level observed at the end of treatment. However, therapeutic effects partially relapsed at 3 months and stabilized at minor LARS levels (score 24) through 1-year follow-up, as shown in Table 3. Symptoms recurred in three dimensions. Regarding bowel movement frequency, the patient reported that daily bowel movements increased from 3 to 4 times after treatment to approximately 5–10 times. A pattern persisted: “If bowel movements were frequent on a given day, the patient would not need to have a bowel movement the following day.” Regarding bowel evacuation disorder and urgency, the patient reported occasional symptom recurrence, and urgency symptoms caused the greatest disruption to daily life. The improvement in liquid stool leakage and stool consistency was well maintained.

**Table 3:** LARS score before and after treatment.

Question	2024-12-05 (baseline)	24-12-11 (3 EA)	24-12-17 (6 EA)	24-12-22 (9 EA)	24-12-27 (12 EA)	2025-02-01 (1 month)	2025-03-28 (3 months)	2025-07-02 (6 months)	2026-01-10 (1 year)
Total score									
	34	27	27	20	11	11	24	24	24
Do you ever have occasions when you cannot control your flatus (wind)?									
	0	0	0	0	0	0	0	0	0
Do you ever have any accidental leakage of liquid stool?									
	3	0	0	0	0	0	0	0	0
How often do you open your bowels?									
	4	2	2	0	0	0	4	4	4
Do you ever have to open your bowels again within one hour of the last bowel opening?									
	11	9	9	9	0	0	9	9	9
Do you ever have such a strong urge to open your bowels that you have to rush to the toilet?									
	16	16	16	11	11	11	11	11	11

The MSKCC-BFI score improved from 51 to 65 after treatment, and the patient’s average daily bowel movements decreased from 15 to 3. The therapeutic effect was primarily manifested in alleviating frequent and urgent bowel movements and improving defecation difficulties. However, no improvement was observed regarding the impact of diet on bowel movements, as shown in Table 4.

**Table 4:** MSKCC-BFI score before and after treatment.

Dimension	Baseline	Following treatment
Total score	51	65
Urgent stool frequency factor	22	33
Diarrhea influenced by dietary factors	16	16
Abnormal defecation sensation factor	13	16
Average daily bowel movements	15	3

The EORTC QLQ-C30 questionnaire indicates that patients experienced improvements in role, emotional, and social functioning following treatment. Symptoms of pain and diarrhea achieved complete remission, and global health increased from 50 to 100, as shown in Table 5.

**Table 5:** EORTC QLQ-C30 score before and after treatment.

Dimension	Baseline	Following treatment
Physical functioning	100	100
Role functioning	33.33	66.67
Emotional functioning	75	91.67
Cognitive functioning	100	100
Social functioning	50	83.33
Fatigue	11.11	11.11
Nausea and vomiting	0	0
Pain	16.67	0
Dyspnea	0	0
Insomnia	0	0
Appetite loss	0	0
Constipation	0	0
Diarrhea	33.33	0
Financial difficulties	33.33	33.33
Global Health	50	100

Qualitative interviews ultimately identified 5 themes comprising 12 subthemes, as shown in Table 6. A brief analysis of the relationship between these findings and quantitative questionnaire results is provided. Since qualitative interviews were used to evaluate the efficacy of acupuncture treatment, we present patients’ descriptions before and after treatment to visually illustrate interview outcomes, as shown in Table 7.

**Table 6:** Qualitative interview results and the relationship with quantitative results.

Theme	Sub-theme	The relationship with quantitative results
Comprehensive improvement in symptoms	Effective alleviation of core LARS symptoms	Triangulation and Complementarity: (LARS; MSKCC-BFI) Presents the patient’s LARS symptoms comprehensively, finds interrelationships among these symptoms, and details the specific manifestations of treatment efficacy.
	Improvement in other symptoms	Triangulation and Complementarity: (EORTC QLQ-C30) Identified the patient’s unique systemic symptoms and the therapeutic efficacy of the treatment targeting these symptoms.
Relief of psychological stress and improvement of life difficulties	Alleviating panic	Complementarity of Breadth and Depth: (EORTC QLQ-C30) Identify the causes of patients’ social, occupational, and daily life impairments, the specific manifestations of psychological stress, and the practical significance of acupuncture treatment for patients’ quality of life.
	Positive transformation in work and life challenges	
Social function and family relationship reconstruction	Re-engagement in social activities	
	The disappearance of the burden of family responsibilities and the return to harmony	
Recognition and recommendations for acupuncture treatment	The sensation of Deqi during EA is intense yet tolerable and is considered an indicator of efficacy.	Exploration and Optimization: Explores patients’ authentic experiences, perceptions, and acceptance of the treatment
	Perceptions of the Time. It takes for acupuncture to take effect	
	Recommendations for treatment plans	
Perceived advantages over other therapies	Recognizing the superior efficacy of EA over other treatments	
	Recognizing the safety and ease of use of EA	
	Strongly oppose ostomy	

**Table 7:** Semistructured qualitative interview results and quote.

Theme	Sub-theme	Before treatment	After treatment
Comprehensive improvement in symptoms	Effective alleviation of core LARS symptoms	*“The urgent symptom is that although I know I have already defecated, I cannot control it and have been using underwear for a long time. I walk with feces.”* *“If I want to go around in the day, I have to circle the bathroom all the way because I have to defecate for less than 10 min.”* *“As soon as I eat or I feel intestinal peristalsis, I have to defecate. I have to go to the bathroom 5 or 6 times while eating.”* *“As soon as I walk, even just getting ready to walk, I have to defecate.”*	*“The situation of controlling bowel movements has improved, from the previous inability to control bowel movements to now being able to control bowel movements about two minutes to ten minutes.”* *“After finishing my meal, I go out for a half-hour run and feel nauseous and down. I can basically hold back my bowel movements until I come back.”* *“I can enjoy my meal without feeling like defecating during meals.”*
		*“I have to defecate almost 10 times per day, with the highest 20 times.”*	*“The frequency of bowel movements has decreased from a dozen times in the past to 3 to 4 times now.”*
		*“Almost every night, I experience leakage of stool.”*	*“During treatment, there was only one time about leakage of stool; it was at night.”*
		*“I always want to defecate when I have a meal, but it is difficult to empty my stool when I go to the toilet.”* *“I have a hard time emptying my bowels, which leads to a frequency of bowel movements, but each time, there is only a bit of feces.”* *“It is difficult to empty my stool, so sometimes I try to have something spicy that hopes it functions like laxatives. Only this allows me to empty my stool.”*	*“Now, it is possible to empty my stool, or I can rub my abdomen to promote emptying. Although it seems that sometimes the frequency of defecation is four or five times, there is no need to defecate for a day after emptying.”*
		*“I used to go an entire year without passing gas。”*	*“Nowadays, i can break wind occasionally, and is not leaking easily.”*
	Improvement in other symptoms	*“I always feel left abdominal distension and pain After eating something.”* *“My hands and feet are cold, and I often sweat.”*	*“Regarding bellyaches, they used to be distension and pain, but now there is no pain. Even though I feel distension, I can relieve it by walking after meals to promote digestion.”* *“Now my hands and feet are not too cold anymore, and I still sweat, but I do not sweat much.”*
Relief of psychological stress and improvement of life difficulties	Alleviating Panic	*“I’m usually afraid of going out, so when I go for a walk, I always go around the toilet. I only dare to go when there are toilets around me.”*	*“Now I am in a good state of mind and can live a normal life after going home.”* *“Now that I’m back home, I can live like a normal person with a positive mindset.”*
	Positive Transformation in Work and Life Challenges	*“My office must be next to the toilet, and I cannot have breakfast because of this situation.”* *“Over the past two years, during follow-up examinations, chronic atrophic gastritis and cholecystitis have gradually appeared.”* *“Every time I go on a business trip, not only do I skip breakfast in the morning, but sometimes I also have to take a pill of loperamide and anti-diarrheal medicine to prevent it affects my driving and making it difficult to find the service area.”* *“The paper rolls bought at home cost thousands of yuan yearly.”*	*“Now that I go home, I can live a normal life with a good mentality and have breakfast if I go to work.”*
Social Function and family relationship reconstruction	The disappearance of the burden of family responsibilities and the return to harmony	*“Because there is not enough toilet at home, my wife and I live in a separate apartment. These things affect the daily lives of family members, cause inconvenience, and trouble them, making their moods more irritable.”* *“My wife is not as enthusiastic as before. She also works and wants to socialize with her friends and colleagues. I feel that she cannot always drag a man with fecal incontinence around her, as she is also troubled by emotions.”* *My wife said: You’re holding everyone back.”*	*“I will not have to fight over the bathroom with my wife and kids, and it will not interfere with my family’s daily life.”* *“I expect to have some collective family life with my wife and daughter and be able to go shopping with them. Previously, my wife went shopping alone.”*
	Re-engagement in Social Activities	*“I could not attend my colleagues’ weddings, funerals, or banquets, so I had to ask my wife to go on my behalf, which also affected my relationships and interactions with colleagues and friends.”*	*“At least I should have no problem drinking tea or having parties with friends. I will not have to run to the bathroom when I have a meal again.”*
Recognition and recommendations for acupuncture treatment	The sensation of deqi during EA is intense yet tolerable, and is considered an indicator of efficacy.	*“I think the principle of acupuncture is the same as sacral nerve treatment. When I accepted the electroacupuncture, there was a slight swelling and a slight wandering, which excited me and gave me hope.”* *“(The feeling of wandering is concentrated around) the anus and in front of the perineum, on both sides of the thighs.”* *“Needling may cause some pain, but compared to incontinence, the pain is not worth mentioning at all. I can tolerate this kind of pain.”*	
	Perceptions of the Time It Takes for Acupuncture to Take Effect	*“After the fourth session (of EA), bowel movements improved. After the ninth session (of EA), I felt I could control my bowel movements.”*	
	Recommendations for Treatment Plans	*“I was wondering if it would be possible to extend this treatment course. Since everyone’s situation is different, some people may find that twelve sessions resolve their issues completely. However, for patients like me with a longer disease duration—nearly 9 or 10 years—extending the treatment course might be more beneficial. This could help patients address their incontinence issues at the root cause.”*	
Perceived advantages over other therapies	Recognizing the superior efficacy of EA over other treatments	*“I think electroacupuncture should be the preferred treatment for my personal efficacy.”* *“During the first four days, it (Sacral nerve treatment) can reduce fecal leakage. After a week, it will return to its previous state, but it will be even more difficult to empty my stool.”* *“There is only one effective point during the first phase of (Sacral nerve) surgery.”*	
	Recognizing the safety and ease of use of EA	*“Sacral nerve treatment requires surgery, which is invasive. and is not safe and effective compared with electroacupuncture.”* *“This (EA) is not as invasive as sacral nerve therapy.”* *“It (EA) is also relatively convenient for sacral nerve therapy. I do not suffer that much.”* *“Fecal microbiota transplantation also requires placing a tube through colonoscopy or gastroscopy for three days, which is uncomfortable. Colonoscopy requires taking laxatives, and gastroscopy requires anesthesia.”*	
	Strongly oppose ostomy	“*Currently, I do not want to become a person with a stoma.”* *“I am unwilling to accept a permanent stoma, no matter what.”*	

## Discussion

LARS is not a short-term postoperative complication and can persist for years or even become lifelong (25). This patient continued to experience LARS 8 years after stoma reversal, significantly impacting quality of life and psychological health, emphasizing the need for early systematic attention to bowel dysfunction and mental health in post-rectal cancer surgery patients. The POLARS score enables preoperative risk assessment of bowel dysfunction based on age, tumor height, stoma status, and preoperative radiotherapy, providing surgeons with predictive insights to optimize postoperative management through early dietary guidance and psychological interventions.

In this case, we selected the Baliao acupoints (BL31–34), which combine traditional and modern anatomical theories. The anatomical location corresponding to the Baliao acupoints is the posterior sacral foramen, through which the sacral nerve passes. Therefore, EA at the Baliao acupoints (BL31–34) may improve LARS by stimulating the sacral nerve and pelvic floor muscle groups. Previous studies have found that EA at Zhongliao (BL33) may facilitate the reinnervation and strengthening of pelvic floor muscles (26). Patient interviews indicate that the feeling of de-qi from EA is similar to that of sacral nerve stimulation, with patients reporting a more pronounced subjective sensation and a longer stimulation duration. From a clinical practice perspective, electroacupuncture also offers advantages such as minimal invasiveness, convenience, and economy. However, this is only a single patient’s anecdotal account and does not constitute evidence for comparing therapeutic efficacy. Nevertheless, this patient’s experience suggests that EA at the Baliao acupoints may be a well-tolerated, easily accessible adjunctive therapy worthy of further exploration for the management of LARS.

LARS is a group of intestinal dysfunction syndromes. In addition to the symptoms reflected in the LARS scale, the timing of abnormal defecation, stool characteristics, and the patient’s defecation rules can serve as the basis for syndrome differentiation and treatment in traditional Chinese medicine, and should be carefully noted. The patient in this report presented with cold extremities, abdominal distension, and urgency-predominant defecation disorders. According to TCM theory, the syndrome differentiation indicated a kidney yang deficiency pattern. Moxibustion at Shenshu (BL23) and Mingmen (DU4) was used to tonify kidney yang.

Several factors limit the interpretation of causality regarding the improvements observed in this case. First, EA, moxibustion, and dietary and lifestyle guidance were delivered concurrently, precluding attribution of the observed changes to any single factor within the intervention protocol. Second, this case report lacked a control condition and blinded outcome assessment, which increases the risk of bias. Third, non-specific contextual effects, including increased clinical attention, therapeutic ritual, and heightened patient expectation during the 24-day intervention period, may have contributed to the initial symptomatic relief. In addition, regression to the mean should be considered explicitly. Given that this patient has an eight-year history of LARS and that symptoms may fluctuate over the long term, the patient may have been experiencing peak symptom severity prior to treatment, rather than a stable long-term average. Therefore, the post-treatment decline in scores may be partly attributable to a statistical regression of extreme measurements toward the patient’s individual mean, rather than a true biological change. However, regression to the mean cannot fully explain the observed changes, as qualitative data also documented clinically significant functional improvements (including longer urgency tolerance and reduced fecal leakage). Regarding follow-up, the patient’s LARS score returned to 24 at 3 months and remained at that level at 6 months and 1 year, suggesting that the patient had entered a new symptom plateau. Since the score did not continue to rise, we cautiously interpret this follow-up pattern as a partial loss of efficacy. The patient reported intermittent home moxibustion during the follow-up period, but no further improvement in the LARS score was observed. Since this intervention lacked standardization, was of low intensity, and was not conducted concurrently with EA, it cannot serve as evidence either supporting or refuting a dose-response relationship. Therefore, insufficient treatment duration or intensity is only one possible explanation for partial relapse, not the primary cause. Other competing hypotheses should also be considered, including the chronic natural fluctuation of the disease, regression to the mean, the waning of the situational effect following the conclusion of intensive home-based interventions, and insufficient adherence to dietary and lifestyle recommendations.

The semistructured qualitative interview with this patient shows us that LARS patients have a variety of psychological and emotional disorders and awkward situations in work, social contact, and family life. Only using the LARS score to evaluate the patient’s situation will ignore the patient’s feelings. A few scholars have used qualitative research to study the emotional experience and social disorders of patients with LARS (27–29). These articles show that LARS disturbed patients’ moods, interrupted their daily activities, and affected their family life. However, the patients felt lonely and were forced to develop coping methods by themselves, which is the same as our outcomes. It is beneficial for the doctor to provide health education to the patient and their family about the incidence, symptoms, and treatment of LARS earlier, so that the patient’s family can help relieve the patient’s negative mood and the patient is not too embarrassed or nervous.

Additionally, a discrepancy was noted between the MSKCC-BFI subscale “Diarrhea influenced by dietary factors” and the patient’s qualitative report of reduced mealtime urgency. The subscale specifically measures sensitivity to certain foods, whereas the patient’s narrative describes improvement in the gastrocolic reflex—an aspect more accurately captured by the “Urgent Stool Frequency Factor”. Thus, the subscale and the narrative are measuring distinct aspects of the patient’s experience, making their findings complementary rather than contradictory. This contrast illustrates the value of mixed-methods research in LARS. In this case, although the patient reported symptomatic improvement after 6 treatments, the LARS score remained unchanged, underscoring the limitations of using a single quantitative instrument to fully describe the clinical course. Thus, quantitative and qualitative data are indispensable for diagnosing and treating patients with LARS. Mixed methods research (30) can combine qualitative and quantitative methods to collect and analyze data, yielding richer, more robust evidence. The author’s team is also trying to apply mixed research to higher-level evidence research on LARS (31).

## Conclusion

This case report comprehensively documented the treatment process of a refractory LARS patient using the LARS scale and a semistructured interview and found that EA combined with moxibustion can provide short-term symptomatic relief and improve the quality of life and mood of patients with LARS. This provides a new idea for treating LARS, but it still lacks support from higher-level evidence.

## Data Availability

The original contributions presented in the study are included in the article/supplementary material, further inquiries can be directed to the corresponding author.
