# Correction: Case Report: Quantitative and qualitative evaluation for electroacupuncture combined with moxibustion in the treatment of recalcitrant low anterior resection syndrome

**DOI:** 10.3389/fmed.2026.1890249

**Published:** 2026-06-12

**Authors:** Chenxi Hu, Xun Li, Lu Yang, Ruolin Fang, Jinchang Huang, Ming Yang

**Affiliations:** 1 The Third Affiliated Hospital, Beijing University of Chinese Medicine, Beijing, China; 2 Centre for Evidence-Based Chinese Medicine, Beijing University of Chinese Medicine, Beijing, China; 3 Department of Acupuncture and Mini-invasive Oncology, Beijing University of Chinese Medicine Third Affiliated Hospital, Beijing, China

**Keywords:** case report, electroacupuncture, low anterior resection syndrome, moxibustion, rectal cancer

The correct corresponding authors are Jinchang Huang and Ming Yang. The corrected Correspondence section appears below:

^*^Correspondence: Jinchang Huang, zryhhuang@163.com; Ming Yang, yangming@bucm.edu.cn

The **Acknowledgments** incorrectly appeared as:

“We appreciated the patient for agreeing to publish the case. We would like to thank Prof. Nicola Robinson, Dr. Lu Yang, and Dr. Ruolin Fang for their suggestions and support of this case report.”

The correct **Acknowledgements** is:

“We appreciate the patient for agreeing to publish the case. We would like to thank Prof. Nicola Robinson for her suggestions and support of this case report.”

The original version of this article has been updated.

